# Distinct Effects of Type I and III Interferons on Enteric Viruses

**DOI:** 10.3390/v10010046

**Published:** 2018-01-20

**Authors:** Harshad Ingle, Stefan T. Peterson, Megan T. Baldridge

**Affiliations:** Department of Medicine, Washington University School of Medicine, St. Louis, MO 63110, USA; hingle@wustl.edu (H.I.); speterson22@wustl.edu (S.T.P.)

**Keywords:** norovirus, rotavirus, reovirus, interferon, microbiota, immunity

## Abstract

Interferons (IFNs) are key host cytokines in the innate immune response to viral infection, and recent work has identified unique roles for IFN subtypes in regulating different aspects of infection. Currently emerging is a common theme that type III IFNs are critical in localized control of infection at mucosal barrier sites, while type I IFNs are important for broad systemic control of infections. The intestine is a particular site of interest for exploring these effects, as in addition to being the port of entry for a multitude of pathogens, it is a complex tissue with a variety of cell types as well as the presence of the intestinal microbiota. Here we focus on the roles of type I and III IFNs in control of enteric viruses, discussing what is known about signaling downstream from these cytokines, including induction of specific IFN-stimulated genes. We review viral strategies to evade IFN responses, effects of IFNs on the intestine, interactions between IFNs and the microbiota, and briefly discuss the role of IFNs in controlling viral infections at other barrier sites. Enhanced understanding of the coordinate roles of IFNs in control of viral infections may facilitate development of antiviral therapeutic strategies; here we highlight potential avenues for future exploration.

## 1. Introduction

Viral pathogens may infect a human host via a variety of routes, including inhalation, oral ingestion, sexual transmission, or the bite of an arthropod. Common to many of these infection routes is that the first encounters between the virus and the host occur at a mucosal surface. These barrier sites are well adapted to respond to pathogens, as they are in constant contact with the outside environment. Interferons (IFNs), first discovered for their ability to “interfere” with influenza virus infection in cell culture, have long been known to be critical molecules of the host innate immune system [1]. IFNs are important for both the first wave of viral regulation, and also for priming of adaptive immune responses [2,3]. It is only over the past decade, however, that we have begun to appreciate differential roles for different IFN subtypes at mucosal surfaces, and indeed that we have been aware of some IFN subtypes. In this review, we will specifically focus on type I and type III IFNs and their unique functions in defending the host against invading viruses.

While it has become clear that both type I and III IFNs have important roles at all mucosal surfaces, the differential functions of these IFNs in viral control have perhaps been most thoroughly explored in the context of intestinal infection. The gut is a complex organ, encompassing a wide variety of epithelial and immune cell subtypes, mucus layers that are distinct in different intestinal compartments, and a diverse community of commensal organisms including bacteria, viruses and fungi. Many functions are distributed along this organ, including digestion of food, absorption of nutrients and water, secretion of waste products, and development and maintenance of a properly tuned immune system [4]. Enteric viruses infecting a host at this complex barrier need to remain intact during passage, bypass or utilize the microbiome and mucus as they approximate to permissive host cells, infect epithelial or immune cells, and ultimately remain localized in the intestine or traffic systemically. 

Here we will review what is known for type I and III IFN control of viral infection in the massively complex compartment of the intestine. We will focus on IFN-induced cell-intrinsic signaling by different IFN subtypes, the specific roles of these IFNs in control of well-studied enteric viruses, interactions between IFNs and the microbiome, and how enteric viruses combat the antiviral effects of IFNs. We will also highlight interesting research directions for type I and III IFNs that may prove important to understanding enteric viral regulation in the future.

## 2. Type I Versus Type III Interferons

As a critical part of the innate immune system’s antiviral response, IFNs have been a topic of immunological and microbiological interest since their discovery nearly 60 years ago [5,6]. This family of cytokines enacts a cellular response stimulating hundreds of IFN-stimulated genes (ISGs), which can have both antiviral and proviral roles [7]. While the three types of IFNs and their receptors are well established, the complex relationships between these cytokines, and precisely how they orchestrate a defense against viral invaders via activity of specific ISGs, are still only partially understood. IFNs are classified into three types (I, II, and III), based upon the cell surface receptor with which they interact. Here we will focus on the critical roles of type I and III IFNs at the gut mucosal interface; type II IFN, or IFN-γ, has been reviewed elsewhere [8]. We will begin with a brief discussion of the inherent differences between type I and type III IFNs.

Type I IFNs are both the first discovered and the largest group within this cytokine family [6]. Identified by their interaction with the ubiquitously expressed IFNαR1/2 heterodimeric receptor [9,10], type I IFNs in humans encompass five types: IFN-ω, IFN-ε, IFN-κ, IFN-β, and IFN-α, for which there are 13 subtypes [11,12,13]. Of these, IFN-α and IFN-β have been the best studied. An important aspect of this large family of proteins is the differential downstream effect they have, mediated through distinct interactions with the same receptor proteins. For example, with a higher binding affinity to IFNαR1, IFN-β causes a more robust antiproliferative effect and higher ISG expression levels than IFN-α [14,15]. Additionally, IFN-β has the unique ability to transduce signals upon IFNαR1 binding without IFNαR2 present [11]. 

Discovered more recently, type III IFNs, or IFN-λ, induce an antiviral response through interaction with a distinct receptor [16,17]. In humans, the four type III IFN proteins include IFN-λ1 (also known as IL-29), IFN-λ2 (IL-28A), IFN-λ3 (IL-28B), and IFN-λ4 [18]. The IFN-λ receptor (IFNλR) is a heterodimeric receptor made up of IFNλR1 (also IL-28Rα) and IL-10Rβ subunits. Type III IFNs have many overlapping functions with type I IFNs, prompting the question as to why synonymous innate immune pathways exist. Insight into the expression of IFNλR1 in specific cell types has suggested a possible reason. While the IL-10 receptor subunit is widely expressed in various cell types, IFNλR1 is predominantly expressed in epithelial tissues [19]. Therefore, its specific utility as a molecular first responder at sites including the lungs and gastrointestinal tract is apparent [20,21].

**Figure 1:**
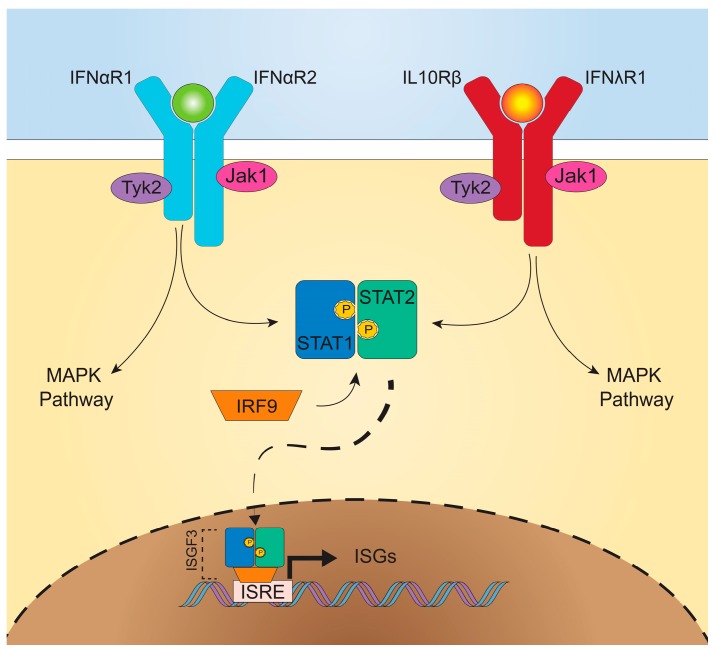
Canonical type I and III IFN signaling. Upon interaction of type I and type III IFNs with their receptors, TYK2 and JAK1 phosphorylation occurs, recruiting STAT1 and STAT2 for phosphorylation. STAT1 and STAT2 dimerize and associate with IRF9 to form the ISG factor 3 (ISGF3) complex, which traffics to the nucleus (dashed arrow) and binds IFN-stimulated response elements (ISRE) to drive transcription of ISGs (bold arrow). Both IFNs also activate the MAPK pathway (not shown). While shown in equivalent quantities here for simplicity, differential type I and type III IFN receptor densities, based on cell type, may influence subsequent host responses.

The signaling pathways downstream from IFNαR and IFNλR are remarkably similar (Figure 1), both inducing activation of multiple signal tranducer and activator of transcription (STAT) proteins and formation of ISG factor 3 (ISGF3) [16]. Upon ligand-receptor binding, IFNαR2- or IFNλR1-associated Janus kinase (JAK) 1 and IFNαR1- or IL-10Rβ-associated tyrosine-specific kinase (TYK) 2, transphosphorylate and phosphorylate associated receptor tyrosine residues [9,22,23]. Recruitment of STAT1 and STAT2 cytosolic proteins allows for their phosphorylation and dimerization, and then the STAT1/STAT2 heterodimer associates with IRF9, forming ISGF3 [24]. Translocation of this complex to the nucleus leads to IFN-stimulated response element (ISRE) binding and promotion of ISG transcription. Both type I and III IFNs signal through this common pathway [25]. Overlapping use of this signaling pathway, as well as shared induction of mitogen-activated protein kinase (MAPK) pathways, demonstrate the similarities in downstream signaling between type I and III IFNs [25,26].

**Figure 2:**
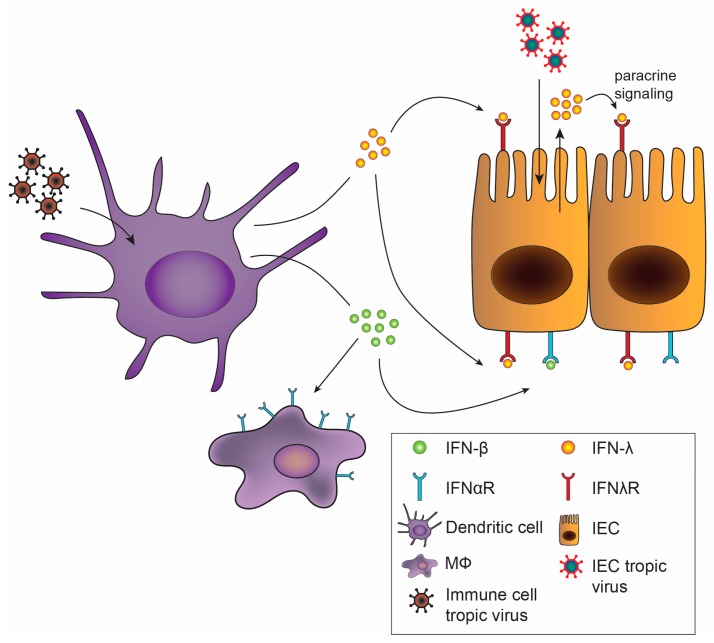
Differential sources of and receptor expression for type I and III IFNs. Immune cell tropic virus sensed by dendritic cells (DCs) results in production of type I and type III IFNs. Type I IFNs activate other immune cells, including macrophages, by binding to IFNαR, and to a limited extent may act on intestinal epithelial cells (IECs). Sensing of IEC tropic virus such as norovirus or rotavirus by IECs may stimulate type I and III IFN production. Type III IFNs, possibly acting by both autocrine and paracrine signaling to neighboring IECs, bind to IFNλR expressed on IECs to induce an antiviral state and protect the host from infection.

Despite use of redundant signaling pathways and similar induction of ISGs between type I and III IFNs [25,26,27], recent studies have identified divergent effects of type I and III IFNs. Depending upon the cell type interrogated, type I and III IFNs may mediate differential expression patterns of ISGs, including distinct gene sets induced in intestinal and respiratory epithelial cells [28,29]. Additionally, type I and III IFNs can exhibit differential kinetics of induction, with IFN-α inducing more rapid but transient ISG expression, while the effects of IFN-λ are delayed but longer lasting [30,31,32]. Alternative regulation of these pathways by negative regulators including suppressor of cytokine signaling 1 (SOCS1) has been implicated in driving some of these differences [33,34]. There is also the possibility that IFN-λ may exhibit unique MAPK signaling pathway activation, not activated by type I IFNs, for non-redundant antiviral activity [35]. In addition to downstream regulation, the production of type I and III IFNs may be differentially regulated. In vitro, type I and III IFNs may be induced in parallel [36,37], but in vivo, preferential induction of IFN-λ at mucosal surfaces by viral infection was observed in both the intestine and the lung [38,39,40], predominantly emanating from epithelial cells. Consistent with this observation, IFN-λ is transcribed and translated at higher rates than IFN-β in intestinal organoids [35]. Activation of an antiviral response in dendritic cells, in contrast, may stimulate production of both type I and III IFNs (Figure 2) [37,41,42], which may act on a variety of cell types.

Of critical importance to the distinct effects of type I and III IFNs in vivo is the differential tissue and cellular expression of their receptors [43]. Type I IFN induces robust ISG responses in many tissues including liver, spleen, and kidney, while type III IFN mediates its most prominent effects on organs with mucosal surfaces [44]. Cell-specific effects on receptor expression have been best described in the gut (Figure 2). Intestinal epithelial cells (IECs) have high expression levels of IFNλR with low levels of IFNαR1 and IFNαR2 [38,45]. The opposite is found in cells of the lamina propria of the gut, with low levels of IFNλR1 and high levels of IFNαR [38]. Increased IEC responsiveness to IFN-λ but not IFN-β due to epithelial cell polarization and differential receptor trafficking further differentiates the roles of type I and III IFNs on the epithelium [46,47]. Therefore, the division between epithelial cells and lamina propria cells confers distinct roles of IFN-λ and IFN-α/β in the gut for protection from initial, early viral infection and systemic spread, respectively [27,38,48]. While recent studies have highlighted distinct potential mechanisms for the disparate antiviral activity of type I and III IFNs observed during infections, future work exploring how these in vitro observations translate to virus-, organ- and cell-specific effects in vivo will be of great interest. We detail the effects of the IFNs in the antiviral response against specific enteric viruses below.

## 3. Differential Control of Individual Viruses by Type I and III IFNs

IFN-mediated control of viral infection differs widely depending upon the virus and its cell and tissue tropism (Table 1). Upon entering the intestinal lumen, a virus can infect IECs, the predominant intestinal cell type, or alternately immune cells in the lymphoid tissue, and from there disseminate to other tissues. Type III IFN mounts an effective antiviral response in IECs because of the unique expression of IFNλR on epithelial surfaces [19]. The gut may benefit from the focused effect of IFN-λ in stimulating an antiviral response exclusively in the barrier cells themselves, thereby avoiding excessive and off-target inflammation of all cell types. While IECs seem to use a predominantly IFN-λ-based defense, cells in the lamina propria and other tissues instead predominantly use type I IFN to prevent systemic infection.

Norovirus (NoV), a ssRNA virus which causes severe gastroenteritis that can be followed by prolonged periods of asymptomatic shedding [49], is an enteric pathogen with differential sensitivity to type I and III IFNs [50]. Many studies have been conducted using a highly effective mouse model, murine NoV (MNoV), for which both acute and persistent strains have been identified [51,52]. Control of infection by acute MNoV strains (e.g., CW3, MNV-1) depends upon the presence of intact type I IFN signaling, as *Ifnar1*^−/−^ and *Stat1*^−/−^ mice succumb to lethal infection [52,53,54,55,56]. Acute MNoV has recently been reported to have a tropism for macrophages, dendritic cells, and B and T cells in the gut, which may explain why type I IFN signaling is important for its control [57]. For control of persistent strains (e.g., CR6), however, type I IFN only prevents spread of virus from its persistent immune-privileged niche in rare IECs [58,59,60] to systemic sites outside of the intestine [61,62], and does not determine viral loads in the intestine. In contrast, IFN-λ has a profound antiviral effect on persistent MNoV strains [21]. *Ifnlr1*^−/−^ mice exhibit elevated levels of virus in intestinal tissues and stool [45,62], correlating with an increase in the numbers of infected IECs [58]. Recombinant IFN-λ prevents and cures persistent enteric MNoV infection [62], mediating its effects through IFNλR1 expression on IECs [45]. While recombinant IFN-λ does not affect acute MNoV infection in wild-type mice [58], IFN-λ has been shown to prevent transmission of acute strain MNV-1 between immunocompromised mice [63]. Thus far, the data points to IFN-λ as critical to controlling NoV infection in IECs, while type I IFNs control viral infection in all other cell types and tissues.

Reovirus is a dsRNA virus that usually causes sub-clinical disease in humans upon respiratory or enteric infection, but which has recently been linked to celiac disease as a potential trigger [75]. It can be readily studied in mice, providing another useful model to explore interactions between enteric viruses and IFNs. Type I IFNs are critical to prevent lethal infection in mice with the T1L strain of reovirus; viral infection induces robust type I IFN production from conventional dendritic cells in Peyer’s patches [48]. Reovirus can also induce type I IFNs in macrophage cell lines, and late stage reovirus infection leads to necroptosis requiring IFN-β production [64]. Interestingly, reovirus is among the pathogens that preferentially activate IFN-λ production instead of type I IFN upon infection, signaling via retinoic acid-inducible gene I (RIG-I)-like receptors (RLR) and peroxisome-associated mitochondrial antiviral-signaling protein (MAVS) to activate type III IFN expression [65]. In the absence of infection, epithelial cell polarization leads to an abundance of peroxisomes that correlate with increased type III IFN expression in human cells [65], predisposing IECs towards type III IFN expression upon infection. This differential induction of one type of IFN over another suggests another level of selective regulation of these innate immune actors. In vivo, IFN-λ controls reovirus levels in the intestine, as IFNλR expression on IECs prevents reovirus from growing to high titers in small intestinal tissues or being shed at high levels in stool [45].

Also in the Reoviridae family, rotavirus is a clinically important enteric pathogen, transmitted via the fecal-oral route, which continues to cause substantial disease in infants in the developing world [76]. Consistent with what was observed for norovirus and reovirus, type III IFN has been shown to be a powerful antiviral effector during rotavirus infection in the intestine [47,66], and type I IFNs help to control systemic spread of infection, indicating a spatial regulation of antiviral defenses [27]. Murine rotavirus infection robustly activates type I IFN and ISG expression in the intestine, the source of induced IFNs primarily being hematopoietic cells as determined by single-cell analysis [77]. Depletion of type I and II IFNs drastically enhances extraintestinal replication of rhesus rotavirus in suckling mice, and a modest increase in infection is observed when type I IFN signaling alone is absent [69]. Exogenous treatment with either type I or type III IFNs restricts infection of rhesus rotavirus in mice [27], and murine rotavirus is successfully cleared in suckling mice with IFN-λ treatment while type I IFN has a more modest antiviral effect [47]. Alone, IFN-λ acts on IECs to induce an antiviral state, but the type 3 innate lymphoid cell-produced cytokine, interleukin-22 (IL-22), is a cooperative factor for optimal ISG induction [67]. More recent studies have provided new insights into rotavirus and IFN interactions. Rotavirus preferentially induces type III IFN responses in human small intestinal enteroids [68], but this type III IFN response does not effectively clear infection, whereas exogenous type I IFN treatment can, suggesting cooperative roles for IFNs from different cell types may be important for viral control. Interestingly, exogenous IFN-β is more protective against rotavirus than IFN-α or IFN-λ, due to faster induction of an antiviral state [68]. Type I and III IFNs cooperate to limit heterologous simian rotavirus infection in mice by inducing a distinct but overlapping set of antiviral genes in IECs [27], with type I IFN being important in neonatal mice but less critical in adult mice, consistent with an age-dependent diminishing responsiveness of IECs to type I IFN [27]. Despite our general consideration of rotavirus as an enteric virus, it is important to remember it can develop into a systemic infection, potentially causing neurological issues and other systemic disease [78]. Our understanding of the coordinate roles of type I and III IFNs in control of rotavirus infection at extraintestinal sites such as the biliary tract is still poorly understood. Thus, better understanding of the important roles that IFNs play in restricting both enteric and systemic infection may help us limit clinical disease severity.

Adenovirus (AdV) is a dsDNA virus that infects both the respiratory and gastrointestinal tracts with the potential to cause serious morbidity and mortality (reviewed in [79]). Children under the age of 5 are most at risk for infection, and present with severe diarrhea and dehydration [80]. Virus can be shed in stool for up to a year following initial infection [81], with the potential for viral reactivation and systemic infection in immunocompromised patients [82]. AdV replicates at high levels in the ileum, though different AdV strains may be distributed along the gastrointestinal tract, and lymphocytes have been shown to harbor the virus, allowing for lengthy durations of viral shedding [83]. Endothelial cells are important for viral sensing via cyclic GMP-AMP synthase (cGAS)-based detection of cytosolic viral DNA, which stimulates type I IFN to drive clearance of AdV [70]. Inhibition of type I IFN enhances AdV growth in fibroblasts [71], and type I IFN administration prevents viral growth in vitro in Caco2 cells [72]. However, whether or not type III IFN affects AdV has not been explored.

Murine cytomegalovirus (MCMV) is a well-studied mouse model for β-herpesvirus. Understanding how the innate immune system controls, or is helpless against, CMV infections is important because of the high seroprevalence of human cytomegalovirus (HCMV) in human populations [84]. Infection in immunocompromised patients can be tissue-specific, usually within the gastrointestinal tract, or systemic, commonly referred to as CMV syndrome [85]. Upon MCMV infection, both type I and III IFNs are upregulated [73,74,86], and IFN-λ effectively contains viral replication of HCMV and MCMV in human and murine IECs, respectively [74]. While IFN-α and IFN-λ are upregulated after infection, IFN-β has been shown to induce a more potent CMV antiviral defense through IRF3 and IRF7-independent pathways [73]. Additional in vivo exploration of the coordinate regulation of MCMV by type I and III IFNs would be of substantial interest for future studies.

## 4. Individual Interferon Stimulated Genes

The IFN response to cellular pathogens stimulates production of a number of cellular proteins, collectively known as ISGs. The antiviral role(s) of each ISG are not yet fully understood because of unique cell type responses [72,87], differential stimulation by different IFNs [33], and antagonistic viral proteins making each ISG:virus interaction unique [88], but also because the antiviral effects of individual ISGs have not yet been adequately explored. Highly variable in their baseline expression, ISGs can be upregulated or downregulated upon IFN stimulation of a cell, or upon direct cell recognition of virus [89,90]. Hundreds of ISGs may play a role in positive or negative regulation of IFN signaling and induction of an antiviral state. The potentially complex relationships between different ISG combinations are another area that has not been adequately explored. Screening methodologies and the advent of CRISPR/Cas9 have facilitated investigations into the role of specific ISGs [7,91,92], but this is an area where much remains to be uncovered. Additionally, much of the study of specific ISGs has been in the context of systemic, not enteric, viral infections. Here we will focus on several ISGs reported to have activity against intestinal viruses.

Ubiquitin-like ISG15 is an ISG with well-understood relationships with other cellular proteins [93]. ISG15 is robustly upregulated in the intestine in response to type I IFNs, and is also induced in IECs by type III IFNs [94,95]. Upon type I IFN-mediated induction, ISG15 is conjugated to a number of cellular proteins, both cytosolic and nuclear, which traverse a wide range of functional roles. This ISG conjugation, or ISGylation, of cellular proteins is important for both antiviral defense and for prevention of excessive inflammation via the RIG-I pathway [96,97,98]. ISGylation has been shown to inhibit the early viral life cycle of MNoV [99]. RIG-I senses dsRNA in the cytoplasm and subsequently stimulates an IFN-mediated response, a cycle that is positively reinforced by induction of more RIG-I by IFN. ISG15 serves to negatively regulate the RIG-I pathway by direct conjugation of RIG-I, thereby preventing excessive inflammatory activation [98]. Thus, ISG15 plays multiple critical roles in IFN responses; future study to clarify its role specifically in other enteric viral infections would be of great interest.

Another specific ISG of interest is IFN-induced protein with tetratricopeptide repeats 2 (IFIT2), also known as ISG54. The role of this protein in IFN-mediated antiviral responses is two-fold: promotion of apoptosis and restriction of translation [100,101]. Stimulated by type I IFN, IFIT2 drives apoptosis via a mitochondrial pathway in coordination with ISG60 [100] and interacts with translation initiation factor eIF3 to restrict translation [101]. IFIT2′s importance for enteric viral control is suggested via study of MNoV, as it is among the targets for viral antagonism by the viral virulence factor 1 (VF1) protein [102]. 

Interferon-induced transmembrane (IFITM) protein 3 is located in the plasma membrane of endosomal vesicles and has been implicated in inhibition of viral entry into the cytosol and reduction of virus infectivity at multiple mucosal surfaces [103]. IFITM3 and related family members were some of the first discovered ISGs, but their specific antiviral roles have only recently been uncovered [104]. IFITM3 does not regulate bacterial or protozoan pathogens [105], but plays a restrictive role against many viruses including reovirus [103,105,106,107]. IFITM3 can prevent viral particle entry into the cytosol by blocking viral fusion to the endocytic membrane [106] or modulating late endosomal compartment function [107], thereby effectively attenuating viral infection. Interestingly, studies of AdV and HCMV have shown that not all viruses that utilize the endosomal pathway for entry are affected by IFITM expression, suggesting viral evasion strategies may be in place to avoid IFITM-mediated restriction [108].

As the number of known ISGs is in the hundreds, we will only focus on one more ISG studied in relationship to enteric viruses. The viperin protein binds the cytosolic side of the endoplasmic reticulum membrane and is heavily upregulated by IFNs [68,109]. Viperin plays a crucial inhibitory role against many viruses, with multiple mechanisms reported, including inhibition of viral replication and egress [109], and activity against mucosal viruses including reovirus [110,111]. Some viruses have developed strategies to hijack viperin, however, including HCMV, which encodes the viral mitochondrial inhibitor of apoptosis protein to traffic viperin to the mitochondria, resulting in decreased cellular metabolism and enhanced infection [112]. Viperin and many other ISGs are involved in the complex antiviral IFN-induced state, and while several have been identified as integral to the response to enteric virus infection, there is still enormous potential for exploration in this area.

## 5. Viral Evasion and Antagonism Strategies

The powerful antiviral signaling stimulated by IFNs has in turn forced viruses to develop a variety of mechanisms to evade these host immune responses. Viruses target innate immune signaling cascades at multiple points, preventing sensing of viral genetic material as foreign to dampen production of IFNs, and also interfering with signaling downstream of IFN receptors (reviewed in [113]). By targeting type I and III IFN signaling, enteric viruses such as rotavirus can combat and elude the host antiviral response. Rotavirus infection leads to degradation of type I and III IFN receptors in vitro and in vivo in infected IECs [114]. The nonstructural protein 1 (NSP1) of rotavirus is a well-studied viral protein that interacts with various host proteins and targets them for proteasomal degradation [115]. NSP1 inhibits IFN signaling by targeting transcription factors IRF3, IRF5 and IRF7 for proteasomal degradation [116,117], as well as other important molecules in the innate immune signaling cascade such as tissue necrosis factor receptor-associated factor 2 (TRAF2), RIG-I and MAVS [118,119,120]. NSP1 also suppresses IFN responses and ISG production by inhibiting activation of transcription factor NF-κB [121], which is critical for inducing transcription of IFN-β and IFN-λ [122], and preventing IFN-mediated phosphorylation and nuclear translocation of STAT1 in vitro [123,124]. The rotavirus structural protein VP3 also inhibits the IFN response [125,126]. VP3 antagonizes the 2′,5′-oligoadenylate synthetase (OAS)/Ribonuclease L pathway that senses cytosolic dsRNA generated during viral replication [127]. Rotavirus has thus developed myriad effectors to counteract the host antiviral response. 

Multiple other enteric viruses also evade the innate immune system through the action of distinct viral proteins. The MNoV VF1 protein, absent in human NoV, localizes to the mitochondria and blocks the expression of *Ifnb*, *Cxcl10*, and *Ifit2* [102]. This VF1-mediated antagonism of type I IFNs correlates with MNoV virulence, as VF1 in naturally attenuated strain MNV-3 does not inhibit the *Ifnb* promoter activation, while VF1 of virulent strain MNV-1 blocks *Ifnb* promoter activity [55,128]. Mammalian reovirus induces a replication-dependent evasion mechanism wherein the viral non-structural protein µNS sequesters IRF3 in the viral replication compartments. This prevents the nuclear translocation of IRF3, thereby resulting in effective inhibition of type I and III IFN production [129]. 

Evasion of cellular sensing pathways for viral infection is a critical mechanism that was observed for a number of pathogens, including several mucosal viruses. Enterovirus coxsackie B virus targets both type I and III IFN pathways by degrading pattern recognition receptor adaptors, such as TRIF and MAVS, by action of viral protease 2Apro, thereby inhibiting IFN production [130]. In the lung, influenza A virus (IAV) targets the stimulator of IFN genes (STING) pathway to evade innate antiviral responses [131]. Fusion peptide, a region of IAV hemagglutinin, helps penetrate the cellular membrane during fusion [132], and abrogates the induction of type I IFNs by fusogenic liposomes [131], a known inducer of antiviral response [133]. The inhibition of IFN responses is thus widely seen across viral infections as an effective method to allow for unperturbed infection of host cells. Further study of these strategies will help to elucidate innate immune pathways and may reveal high-yield druggable targets for viral infection. 

## 6. Physiological Effects of IFNs on the Intestine 

**Figure 3:**
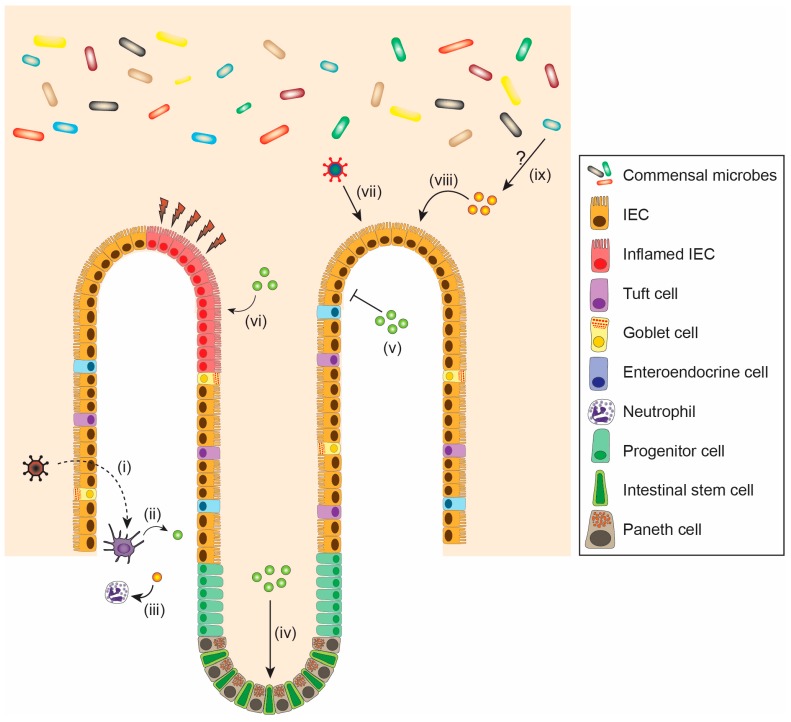
Pleiotropic effects of IFNs on the intestine. (i) Sensing of viral pathogens by dendritic cells (dotted arrow) leads to the production of (ii) type I and III IFNs [37]. (iii) Type III IFNs act on (solid arrow) neutrophils to suppress tissue damage and protect against fungal pathogens [139]. Type I IFNs (iv) inhibit intestinal stem cell proliferation [86], (v) prevent (T bar) systemic viral infection [54] and (vi) contribute to repair of acute tissue damage and wound healing (red lightning symbols) [140]. (vii) IEC tropic virus induces the production of type III IFNs by IECs [47]. (viii) Type III IFNs are crucial in controlling intestinal viral infections [45]. (ix) Commensal microbes may interact with type III IFNs in the gut, though the exact mechanism is unknown [61].

In addition to the well-known role of IFNs in antiviral defense, these signaling proteins play a crucial role in maintaining intestinal homeostasis and cellular proliferation during infection, inflammation, or repair of the intestinal epithelium (reviewed in [134,135,136]) (Figure 3, Table 2). The epithelial cell lining in the intestine provides both a chemical and physical barrier, allowing for maintenance of gut integrity and mucosal homeostasis. The intestinal epithelial barrier consists predominantly of IECs including enterocytes as well as multiple specialized cellular subtypes such as Paneth cells, goblet cells, tuft cells, and enteroendocrine cells [137,138]. 

Type I IFNs, which are constitutively produced in the small intestine [141], appear to play complex roles in regulating IECs under both homeostatic and non-infectious stress conditions [142]. Deletion of type I IFN receptor *Ifnar1* specifically in IECs has been reported to result in an increased number of Paneth cells and epithelial hyperproliferation [143], associated with an increased propensity to colitis-induced tumorigenesis. Another study indicated that IEC proliferation is unchanged in mice lacking *Ifnar1* in all cell types [144]. These reports suggest the potential for context-dependent effects of type I IFN on the intestinal epithelium at baseline. 

During a non-infectious challenge, type I IFNs appear to have the potential to protect or harm the host, mediated through anti-proliferative effects on the epithelium. In the context of excessive β-catenin activation, which leads to intestinal hyperplasia and loss of barrier function, type I IFNs control the proliferation and function of the intestinal epithelium and maintain the barrier [145]. Similarly, when IFN-β production is upregulated with DNA damage, the resulting activation of the p53 pathway promotes senescence in vitro and inhibits intestinal stem cell proliferation in vivo [146]. Elevated levels of type I IFNs can contribute to repair of acute tissue damage associated with graft-versus-host disease [147,148]. With a colitis-related challenge, type I IFNs also have protective effects during acute intestinal damage [140,144,149]. Paradoxically, however, type I IFNs inhibit the resolution of inflammation after injury [144], and treatment of inflammatory bowel disease (IBD) patients with type I IFNs has resulted in mixed outcomes [150]. Chronic upregulation of type I IFNs as occurs with viral infection such as MCMV, or in mice lacking *Irgm1*, has been reported to play a protective role during wound healing [86]. Interestingly, and in contrast to other reports, this protective effect is via proproliferative signaling on the intestinal epithelium, mediated via IFNαR1 on nonepithelial cells including macrophages. In sum, type I IFN signaling plays vital roles in maintenance of intestinal barrier function and protection, but these roles depend strongly upon context, and may include distinct effects on different cell types that cumulatively are protective or damaging depending upon the magnitude of IFN signaling.

Recent studies have revealed that type III IFNs also appear to play important roles in the intestine outside of viral responses. Patients with IBD exhibit increased levels of IFN-λ and IFNλR in intestinal biopsies [151], with IFN-λ being derived from dendritic cells in the lamina propria and IFNλR restricted to IECs. Treatment of patient-derived intestinal organoids with IFN-λ resulted in STAT1 phosphorylation and promoted epithelial proliferation [151]. In vivo, several studies have implicated type III IFN as protective in colitis models. Mice lacking *Ifnlr1* have been reported to exhibit enhanced colitis-related pathology [151,152,153], and IFN-λ treatment has been reported to contribute to wound healing [151]. Activity of type III IFNs on neutrophils has been implicated in this protection, as neutrophils express IFNλR and treatment with IFN-λ decreases neutrophil degranulation, thereby suppressing neutrophil-dependent tissue damage [153]. One study also suggested type I IFN signaling was dispensable for colitis protection in the context of type III IFN-deficiency, as *Ifnar1^−/−^Ifnlr1^−/−^* mice phenocopied *Ifnlr1^−/−^* mice [152]. Thus, both type I and III IFNs may play overlapping or coordinate roles in protecting the intestine during non-infectious challenges and in homeostasis. Further work to dissect precisely how these pathways interact to maintain intestinal health outside of the context of infection will be important in considering their roles in treatment for IBD and other intestinal diseases.

## 7. IFNs and the Microbiota 

While many sites in the human body are colonized by communities of microbes, the microbiota of the intestinal lumen represents one of the densest and most diverse compilations of bacteria, fungi, viruses, protozoa and archaea [154,155]. The microbiota is non-uniform along the gastrointestinal tract, with a complex biogeography and substantial variation along both the longitudinal and transverse axes [156]. Commensal bacteria have been implicated in facilitating infection by multiple enteric viruses, including poliovirus, reovirus, rotavirus, and NoV [61,157,158,159], but conversely also in preventing or controlling systemic viral infection or infection at other mucosal sites such as the lung [160,161]. Here, we will describe what is understood thus far about interactions between the microbiota, IFNs, and viral infections.

The microbiota has been reported to have profound effects on type I IFN-mediated antiviral immunity. Mice administered oral antibiotics exhibit defective clearance of both systemic lymphocytic choriomeningitis virus and influenza infections, and macrophages from these mice are impaired in their type I IFN responses to infection [160]. A recent report implicates microbial metabolite desaminotyrosine as a critical regulator of type I IFN and protection against influenza, indicating this may be a key microbiota mediator for antiviral protection [162]. Intriguingly, infection with enteric helminth *Heligmosomoides polygyrus* has also been reported to stimulate antiviral effects against respiratory syncytial virus infection dependent on both the microbiota and type I IFN signaling [163]. It is possible that some of these microbiota-driven effects on type I IFNs are mediated through levels of plasmacytoid dendritic cells, which produce type I IFNs in response to viral infection but are substantially depleted in the gut in the absence of the microbiota [164].

In addition to these systemic innate immune phenotypes, the local physiological effects of type I IFNs described above may be driven at least in part by components of the microbiota. The Paneth cell expansion and epithelial hyperproliferation reported in mice lacking *Ifnar1* in IECs was microbiota-dependent, as it disappeared when *Ifnar1*-deficient and *Ifnar1*-sufficient mice were cohoused [143]. Removal of endogenous enteric viruses by treatment of mice with an antiviral cocktail makes mice more susceptible to damage with a colitogenic agent [165], a protective effect associated with TLR3- and TLR7-induced type I IFN production. Intriguingly, a separate study confirmed that depletion of enteric viruses augmented tissue damage in wild-type but not in *Ifnlr1^−/−^* mice, suggesting instead that enteric viruses require IFN-λ signaling to protect the host from developing intestinal inflammation [153], indicating that enteric viruses may potentially affect both type I and III IFN signaling. Of interest, depletion of the microbiota enhances intestinal injury and pathogenic bacterial infections in wild-type mice, but these phenotypes can be rescued by infection of antibiotics-treated or germ-free mice with persistent MNoV [166]. This rescue by MNoV requires intact type I IFN signaling, consistent with a protective role for this cytokine family.

There have been a limited number studies exploring the interactions between the microbiota, IFNs, and enteric viruses. Infection of mice by persistent MNoV strain CR6 was found to be enhanced by the presence of intestinal commensal bacteria, a dependence that was abrogated in mice lacking *Ifnlr1*, but not *Ifnar1* [61]. These findings suggest a role for type III, but not type I, IFN in regulating interactions between enteric viruses and the intestinal microbiota. Nucleotide-binding oligomerization domain-like receptor 6 (NLRP6) plays a critical role in the inflammasome, influencing the production of proinflammatory cytokines [167]. NLRP6, which is important for induction of antiviral type I and III IFN responses to MNoV and viral control, is also important for maintenance of gut microbiota homeostasis [168], supporting the idea of coordinate regulation of host responses by the microbiota and viral pathogens. In exploring other mucosal surfaces, it was recently reported that activation of type III IFNs in response to IAV infection altered the upper airway microbiome and increased susceptibility to infection by bacterial pathogens [169]. Additionally, in the lung type I and III IFNs have been found to act coordinately to mediate neutrophil antifungal responses [139]. Extrapolating these results to the intestine, they raise interesting possibilities for alteration of the intestinal microbiota by type III IFNs and/or enteric viral infection. MNoV infection has been reported to alter the intestinal commensal bacteria in some studies but not in others, possibly due to facility or viral strain differences [170,171], and human NoV infection is associated with dysbiosis in a subset of patients [172]. In human pediatric patients severe acute gastroenteritis, especially with rotavirus infection, significantly reduces intestinal microbial diversity [173], supporting the notion that enteric viral infection may regulate the bacterial microbiome.

While studies thus far indicate that commensal bacteria promote type I IFN-mediated antiviral signaling with potent extraintestinal effects, the interactions between type III IFNs, enteric viruses, and the microbiota are less clear. Future opportunities for study include exploring the effects of enteric viruses on the microbiota in *Ifnlr1*-sufficient and -deficient mice, determining how bacteria and viruses influence induction of IFNs by each other, and careful delineation of specific microbiota factors that promote or prevent viral infections.

## 8. Type I and III IFNs at other Barriers 

Despite different cell types and infectious challenges, type I and type III IFNs are also crucial in controlling antiviral responses at other barrier sites beyond the gut [134,135]. In the lung, IFN-λ is rapidly induced prior to type I IFNs after IAV infection, providing early antiviral protection against sublethal IAV infection [174]. However, for long-term protection both type I and III IFNs are necessary. Type III IFNs induce a prolonged antiviral immune response in neutrophils without activating inflammatory cytokines, whereas type I IFNs predominantly initiate a neutrophil-driven inflammatory signature during IAV infection [174]. Therapeutically, treatment with IFN-λ during IAV infection in mice ameliorated disease and reduced mortality as compared to IFN-α. This contrasting effect was due to the rapid induction of proinflammatory cytokines in immune cells and apoptosis in airway epithelial cells by IFN-α but not IFN-λ [175]. Thus, distinct activities of type I and III IFNs are not observed exclusively in the gut but also at other mucosal surfaces. 

Type I and III IFNs also play pivotal roles in controlling feto-placental infections such as during Zika virus (ZIKV) infection, recently shown to be a concerning cause of fetal abnormalities [176,177,178]. Deficiencies in type I IFN signaling, either via *Ifnar1*-deficiency or deficiency of *Irf3*, *Irf5*, and *Irf7* such that little IFN is produced, have been key to modeling ZIKV infection in mice and recapitulating aspects of human disease, supporting a critical role for type I IFN in controlling ZIKV infection [179,180,181]. Type I IFN of fetal origin might provide partial protection to ZIKV infection [182]. Type III IFNs also appear to be critical for viral control. Primary human trophoblasts, the barrier cells of the placenta, are resistant to ZIKV infection, producing high basal levels of type III IFNs that act in autocrine and paracrine manner to restrict ZIKV infection [183]. Despite high basal expression, however, ZIKV infection does not induce type III IFNs in these trophoblasts [183]. Cell-line-based models of human syncytiotrophoblasts also exhibit robust type III IFN production [184]. Pretreating pregnant *Ifnar1*^−/−^ mice with IFN-λ during mid-gestation suppresses ZIKV and alleviates fetal growth restriction, suggesting this as a promising treatment strategy for infection [182]. 

Along with a direct antiviral role, IFN-λ also restricts viral infection by modulating endothelial barriers such as the blood brain barrier (BBB) for protecting against CNS infections. IFN-λ limits West Nile Virus spread to the CNS by tightening the BBB [185]. *Ifnlr1^−/−^* mice showed increased BBB permeability after infection, and IFN-λ treatment enhances colocalization of endothelial junction proteins, ZO-1 and claudin-5 to increase BBB tightness [185]. In contrast, type I IFNs are required for control of viremia and cellular tropism in West Nile virus infection, as infection of *Ifnar1^−/−^* mice results in a rapidly fatal infection associated with high viremia [186,187], a phenotype recapitulated in mice specifically lacking *Ifnar1* only in myeloid cells [188]. A similar set of observations has been reported with yellow fever virus vaccine strain YFV-17D. Type III IFNs prevent spread of YFV-17D to the CNS, as shown by increased infection susceptibility and BBB permeability in *Ifnar1^−/−^Ifnlr1^−/−^* mice compared to *Ifnar1^−/−^* mice [189]. However, type I IFNs are also critical for control of YFV-17D, as robust systemic infection is only achieved in the absence of *Ifnar1* [189].

The recurring theme of sometimes overlapping but distinct antagonistic effects of type I and III IFNs against viral infections has thus been observed in the gut but also at multiple barrier sites throughout the body. Type III IFNs are critical for protecting at the barrier sites themselves, while type I IFNs induce robust systemic responses whenever an invading virus manages to move past the barrier.

## 9. Conclusions and Future Directions

Recent studies of the control of viral infection by type I and III IFNs has revealed distinct roles for these cytokines in the gut and at other barrier surfaces. Due to its more restricted and less systemic inflammatory activity, the potential for IFN-λ administration as a therapeutic intervention in the case of severe or persistent enteric or mucosal infections is clear. In mouse models, IFN-λ has been shown to have potent activity against NoV, rotavirus, and influenza without accompanying damaging inflammation [45,47,62,175], and in humans, IFN-λ has been shown to be safe for administration [190]. Initially explored as a potential treatment for chronic hepatitis C infection, the promise of IFN-λ for treatment of mucosal viruses in humans has not yet been tested but is an exciting potential future direction. Further discovery of both how the microbiota regulates IFNs and viral infection in the intestine is critical to helping inform development of probiotic or metabolic approaches to prevent or treat infections and enhance vaccine responses. In addition to the profound effects of the microbiota on enteric viruses in animal models, there are indications that intestinal bacterial populations may influence responses to oral rotavirus vaccines in humans, though the mechanisms are not yet well understood [191,192]. Continued exploration of the role of commensal bacteria in determining innate and adaptive immune responses to viral challenges will be important to combatting viral epidemics and ensuring development of robust antiviral immunity. Finally, there are still broad opportunities to better understand the specific ISGs induced by both type I and III IFNs and how they target viruses. While there have been a number of successful screens conducted exploring the effects of human ISGs against viruses including West Nile virus and yellow fever virus [7], these methods have not yet been applied to explore the role of specific ISGs in control of enteric viruses. In addition, the ongoing identification of type III IFN-specific ISGs in responsive cell types has the potential to increase the range of interesting ISGs to test in future assays [28]. In conclusion, though the past two decades have revealed an enormous amount about the coordinate control of mucosal viruses by different IFNs, there are still many unanswered questions remaining and exciting avenues left to explore.

## Figures and Tables

**Table 1:** Differential effects of type I and III IFNs on enteric viruses.

Virus	Type I IFN	Type III IFN	References
Norovirus (NoV)	Prevents lethality from acute MNoV infection Controls systemic spread of persistent MNoV	Controls persistent intestinal infection by restricting IEC tropism Therapeutic administration clears persistent MNoV and prevents transmission of acute strain Associated with interactions between microbiota and NoV	[45,52,54,56,58,61,62,63]
Reovirus	Robustly induced by infection in vitro Prevents lethal infection in vivo	Preferentially induced in some cell types Controls intestinal levels and shedding into stool	[45,48,64,65]
Rotavirus (RV)	Treatment decreases local spread and replication in intestinal enteroids Controls diarrhea and systemic replication in vivo Combined deficiency of IFN-α and IFN-γ signaling increases mortality in suckling mice Postnatal mice are responsive to IFN-β treatment, but this diminishes with age	Robustly induced in human enteroids Controls infection in the intestine, acting synergistically with IL-22, and can effectively treat infection in mature mice	[27,47,66,67,68,69]
Adenovirus (AdV)	Induced by in vitro infection Antiviral when administered in vitro	Unknown	[70,71,72]
Murine cytomegalovirus (MCMV)	Induced by infection IFN-β has most effective antiviral effect of type I IFNs	Induced by infection Robust antiviral effects against MCMV	[73,74]

**Table 2:** Differential effects of type I and III IFNs on intestinal physiology.

Challenge	Type I IFN	Type III IFN	References
Inflammation and colitis	Repairs acute intestinal damage associated with colitis and graft-versus-host disease Inhibits resolution of inflammation	Protects in colitis Suppresses neutrophil-dependent tissue damage	[140,144,147,148,149,151,152,153]
Injury repair	Controls intestinal stem cell proliferation and maintain function of the intestinal epithelium Maintains epithelial barrier Facilitates wound healing	Contributes to wound healing	[86,145,146,151]
Homeostasis	Keeps number of Paneth cells and epithelial proliferation in check	Promotes epithelial proliferation in vitro	[143,151]
